# The Association of Geographic Coordinates with Mortality in People with Lower and Higher Education and with Mortality Inequalities in Spain

**DOI:** 10.1371/journal.pone.0133765

**Published:** 2015-07-24

**Authors:** Enrique Regidor, Laura Reques, Carolina Giráldez-García, Estrella Miqueleiz, Juana M. Santos, David Martínez, Luis de la Fuente

**Affiliations:** 1 Department of Preventive Medicine and Public Health, Faculty of Medicine, Universidad Complutense de Madrid, Madrid, Spain; 2 Instituto de Investigación Sanitaria del Hospital Clínico San Carlos IdISSC, Madrid, Spain; 3 CIBER Epidemiología y Salud Pública CIBERESP, Madrid, Spain; 4 National School of Public Health, Instituto de Salud Carlos III, Madrid, Spain; 5 Departament of Sociology, Universidad Pública de Navarra, Pamplona, Spain; 6 National Epidemiology Center, Instituto de Salud Carlos III, Madrid, Spain; Geisel School of Medicine at Dartmouth College, UNITED STATES

## Abstract

**Objective:**

Geographic patterns in total mortality and in mortality by cause of death are widely known to exist in many countries. However, the geographic pattern of inequalities in mortality within these countries is unknown. This study shows mathematically and graphically the geographic pattern of mortality inequalities by education in Spain.

**Methods:**

Data are from a nation-wide prospective study covering all persons living in Spain's 50 provinces in 2001. Individuals were classified in a cohort of subjects with low education and in another cohort of subjects with high education. Age- and sex-adjusted mortality rate from all causes and from leading causes of death in each cohort and mortality rate ratios in the low versus high education cohort were estimated by geographic coordinates and province.

**Results:**

Latitude but not longitude was related to mortality. In subjects with low education, latitude had a U-shaped relation to mortality. In those with high education, mortality from all causes, and from cardiovascular, respiratory and digestive diseases decreased with increasing latitude, whereas cancer mortality increased. The mortality-rate ratio for all-cause death was 1.27 in the southern latitudes, 1.14 in the intermediate latitudes, and 1.20 in the northern latitudes. The mortality rate ratios for the leading causes of death were also higher in the lower and upper latitudes than in the intermediate latitudes. The geographic pattern of the mortality rate ratios is similar to that of the mortality rate in the low-education cohort: the highest magnitude is observed in the southern provinces, intermediate magnitudes in the provinces of the north and those of the Mediterranean east coast, and the lowest magnitude in the central provinces and those in the south of the Western Pyrenees.

**Conclusion:**

Mortality inequalities by education in Spain are higher in the south and north of the country and lower in the large region making up the central plateau. This geographic pattern is similar to that observed in mortality in the low-education cohort.

## Introduction

Geographic patterns in mortality are widely known to exist in many countries. For example, the regions with the highest mortality rates are found in the north of the United Kingdom [1], in the south-eastern United States [2], in northern France [3], in the north and some areas of the south in Italy [4], in the south of the Netherlands [5] and in south-western Spain [6]. The pattern observed in each of these countries basically reflects the geographic distribution of mortality from cancer and/or cardiovascular diseases, since these represent the two leading causes of death [7–8]. For this reason, in countries with a north-south or west-east pattern, the geographic coordinates of latitude, in the first case, and of longitude, in the second case, show a relation to total mortality and to mortality from some of these causes of death [9–11].

However, with the exception of one study carried out in Italy [12] and another in the United States [13], the geographic pattern of socioeconomic inequalities in mortality within these countries is unknown. If geographic patterns in mortality in lower and higher socioeconomic groups are different, as occurs in Italy, the magnitude of mortality inequalities will vary across geographic areas [12]. If, on the other hand, mortality in socioeconomic groups shows a similar geographic variation, the variation in the magnitude of inequalities is minimal, as occurs in the United States [13].

The present study aims to add to the empirical evidence by showing mathematically and graphically the relation of latitude and longitude to total mortality and to mortality from the leading causes of death in groups with lower and higher education in Spain, as well as the geographic pattern of mortality inequalities by education.

## Methods

The data source was a national prospective study including all people in the 2001 census, who were followed up for 7 years and 2 months to determine their vital status. The data were prepared by the National Institute of Statistics based on individual census records linked with the population and mortality registries using common identifiers. Deaths refer to persons who died between 1 November 2001 –the date of the census–and 31 December 2008 –the end of the follow-up period. The National Institute of Statistics provided the investigators with the final data file after removing personal information to maintain confidentiality. The final cohort was composed of 40,148,305 individuals, excluding the 1.7% of subjects who could not be found in the population or mortality registries. The contribution of 395,675 persons to the risk of death was censored because they had moved out of Spain and information could not be obtained thereafter.

This study included the 28,944,854 subjects who were 25 years of age or older on 1 November 2001, since persons of this age would most likely have completed their highest level of education. After excluding 0.8% of subjects due to lack of information on education, 196.5 million person-years at risk and almost 2.4 million deaths distributed in the 50 Spanish provinces were analysed. The province of residence is the maximum geographic disaggregation contained in this data source.

The study subjects were divided into two cohorts for the analysis: a cohort of subjects with low educational level, which includes individuals whose level of education was the first cycle of secondary education [10^th^ grade in the US] or less education, and another cohort of subjects with high education level, which includes individuals whose level of education was the second cycle of secondary education [12^th^ grade in the US] or university studies [college, master and doctorate]. Data for latitude and longitude were obtained from Google Maps. Each province was assigned the latitude and longitude value of its capital city. The two provinces of the Canary Islands are located off the north-western coast of Africa, near southern Morocco. These provinces were assigned the same latitude as Cadiz and the same longitude as Pontevedra, the southernmost and westernmost provinces of continental Spain, respectively, to avoid distorting the estimates due to their geographic coordinates, which are very different from those of continental Spain.

In each province, age- and sex-adjusted mortality rates from all causes were estimated for each of the two cohorts, using the European standard population. By using the method of direct standardization age-specific mortality rates are stratified by sex and are applied to the standard population stratified by sex. For each age stratum the expected number of deaths is the sum of the expected number of deaths for males plus the expected number of deaths for females in that stratum. Finally, the sum of deaths in each age stratum is divided by twice the total for the standard population to obtain the age- and sex-adjusted mortality rates. For the geographic representation of these estimates, the mortality rates were classified into three categories using the Jenks index [14–15], also called “natural breaks”; this minimises the intraclass variation and maximises the interclass variation between distribution categories, so that the distribution of the intervals in each category is as similar as possible to the distribution of the observations in natural clusters.

Age- and sex-adjusted mortality rates from the leading causes of death–cancer, cardiovascular, respiratory and digestive diseases–were also estimated for each of the two cohorts of subjects in each province. In each cohort, the relation between the two geographic coordinates and mortality from all causes and from the leading causes of death was evaluated by calculating the regression coefficients and the linear correlation coefficients. To test whether the data deviated from linearity, we also estimated quadratic regression models.

It is common to assume that observations at sites near each other tend to have similar values due to similarity in several characteristics. Spatial autocorrelation in the residual errors can cause inefficient estimation of the standard regression model parameters and inaccuracy of the variance and significance tests. To evaluate the possible spatial autocorrelation of the data we estimated a mixed linear model. We added an autoregressive term to the predictor variables. Specifically we added the average value of mortality from all nearby provinces. This model is known as simultaneous autoregressive [SAR]. The province structure is represented mathematically by a spatial weights matrix, W. This is a binary 50 x 50 matrix, where the off-diagonal elements, W_*ij*_ for *i* ≠ *j*, equal one if province *j* is a neighbour of province *i* and zero otherwise. In the final weights matrix that is used in the model the elements in each row are standardized so that they sum to one. Analyses were performed with SAS version 9.2.

The centrally located provinces had a higher proportion of the population residing in rural areas. Since the risk of mortality has been shown to be lower in rural than in urban areas [16], we calculated for each province the percentage of the population aged 25 years and over with low and with high education who resided in municipalities with fewer than 5000 inhabitants. This was done by accessing the 2001 population census data in the website of the National Institute of Statistics [17]. We then investigated the degree to which rurality could explain the possible relation between geographic coordinates and mortality in the two cohorts, by including the measures of rurality in the regression models.

The mortality rate ratio for all-cause death was then calculated in each province, taking the cohort of subjects with high educational level as the reference category. These estimates were also represented graphically using the Jenks index [14–15]. Finally, since the latitude was the only geographic coordinate that showed a significant relation to mortality in the regression models in the two cohorts, we calculated the magnitude of socioeconomic inequalities in mortality in different latitudes, by comparing mortality rate in subjects with low versus high educational level. Specifically, the 50 provinces were grouped into quintiles according to value of latitude and in each quintile we estimated the mortality rate ratio in the cohort of subjects with low educational level versus those with high educational level.

### Ethics statement

This study was approved by the Institutional Review Board of National Institute of Statistics. Our database did not include individual identifiers, and consequently the approval by the Ethics Committee was not required.

## Results

**Fig 1:**
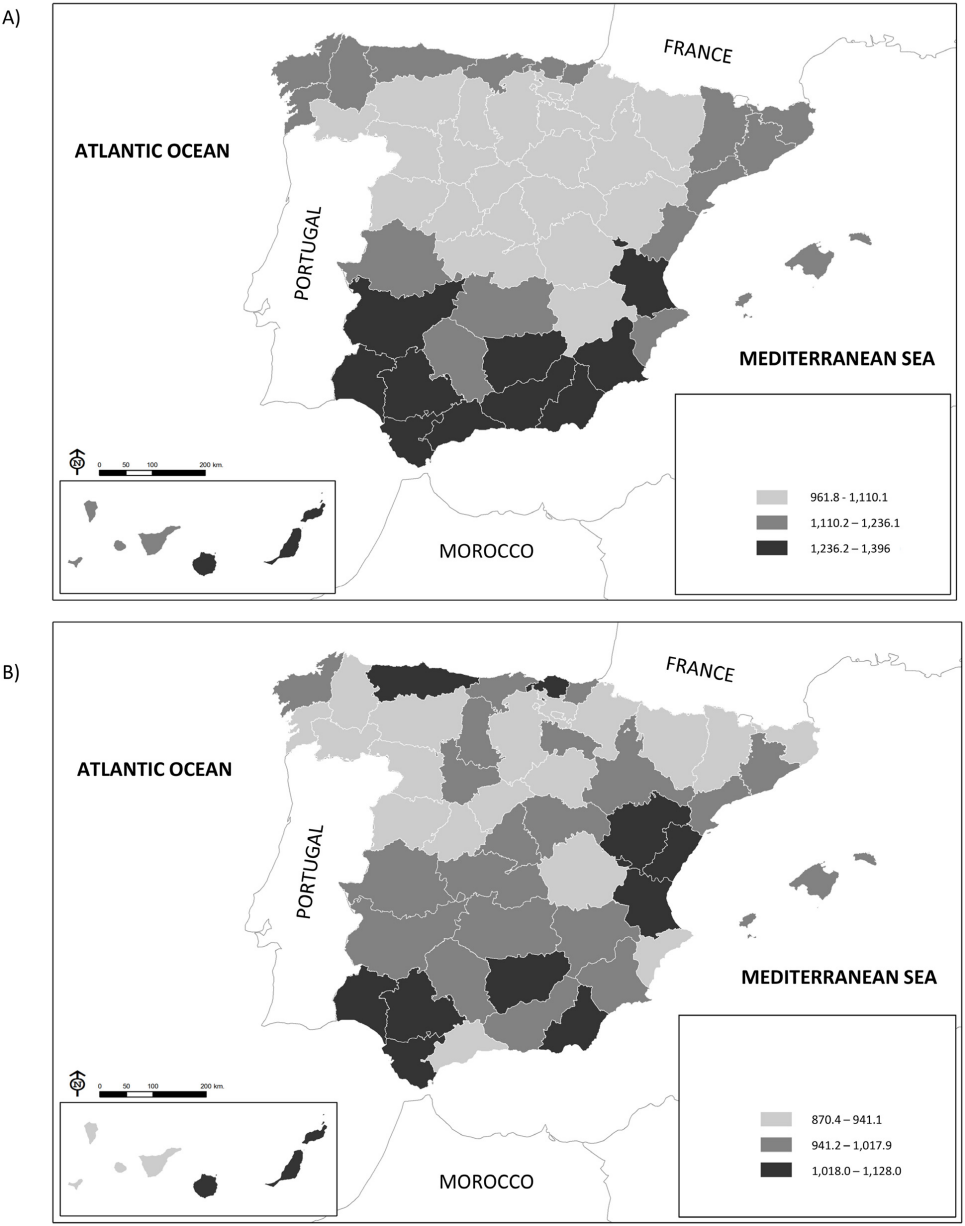
Age- and sex-adjusted mortality rates per 100,000 person-years at risk, by province, in A] the low education cohort and B] the high education cohort. Spain 2001–2008.

The characteristics of the study population and the mortality rate by province of residence are shown in S1 Table. The maps in Fig 1 show all-cause mortality rates by province in the cohorts of subjects with low and high education, respectively. In the cohort with low education, the highest rates are seen in the southern provinces, while intermediate rates are observed in the provinces of the north, and the lowest rates are seen in the central provinces and those located in the south of the western Pyrenees. In the cohort with higher education, the geographic pattern of rates is not as clear: there is a less pronounced concentration of the highest rates in the southern provinces, as high rates are also seen in some eastern and northern provinces, and intermediate rates are also observed in some central provinces, as well as in some provinces in the north, east and south.

The regression and linear correlation coefficients were significant only for latitude, except for cancer mortality in subjects with low education and mortality from respiratory and digestive diseases in subjects with high education (S2 Table and Table 1). Table 1 shows the correlation coefficients of the two geographic coordinates with mortality rates in the two study cohorts. The correlation coefficient between latitude and the all-cause mortality rate was -0.62 in the cohort of subjects with low education and -0.39 in those with high education. The correlation coefficients between latitude and the mortality rates for cardiovascular, respiratory and digestive diseases were also negative in both cohorts. In contrast, the correlation coefficients for the relation between latitude and cancer mortality rates were positive.

**Table 1:** Pearson correlation coefficient for provincial latitude and provincial longitude with age- and sex-adjusted mortality rate in subjects with low and high education for all causes and for leading causes of death. Spain, 2001–2008.

Cause of death	Latitude				Longitude			
	Low education cohort		High education cohort		Low education cohort		High education cohort	
	Coefficient	p value	Coefficient	p value	Coefficient	p value	Coefficient	p value
All causes	-0.62	<0.001	-0.39	0.006	-0.17	0.245	-0.03	0.821
Cancer	0.12	0.410	0.41	0.003	-0.26	0.072	-0.14	0.323
Cardiovascular diseases	-0.60	<0.001	-0.43	0.002	-0.18	0.217	-0.23	0.116
Respiratory diseases	-0.43	0.002	-0.26	0.072	-0.17	0.228	-0.14	0.313
Digestive diseases	-0.60	<0.001	-0.26	0.070	-0.04	0.795	0.03	0.858

The quadratic regression models did not improve the adjustment obtained with simple linear regression, except for the relation between latitude and mortality rates in the cohort of subjects with low education, which showed a significant increase in the variance explained (S3 Table). Table 2 shows the magnitude of the R-square coefficients of the simple linear regression and the quadratic regression, and the statistical significance of the change in the magnitude of the coefficients. In the cohort of subjects with low education, the change in the magnitude of all the coefficients was significant.

**Table 2:** R-Square from regression models predicting provincial sex and age-adjusted mortality rate in each study cohort for all causes and for leading causes of death. Spain, 2001–2008.

	Low education cohort			High education cohort		
	Model 1	Model 2	p value for change	Model 1	Model 2	p value for change
All causes	0.383	0.587	<0.001	0.149	0.157	0.536
Cancer	0.014	0.259	<0.001	0.166	0.166	0.948
Cardiovascular disease	0.359	0.485	0.001	0.185	0.242	0.066
Respiratory disease	0.179	0.253	0.037	0.066	0.112	0.124
Digestive disease	0.356	0.469	0.003	0.067	0.097	0.224

Model 1 includes a single independent variable: latitude. Model 2 includes two independent variables: latitude and latitude*latitude.

The comparison of estimates using a standard regression (independence model) and using an autoregressive model (simultaneous autoregressive model) is shown in S4 Table. Both models were compared using the Likelihood Ratio Test and no spatial autocorrelation was detected. Both models fit the data in a similar way. Both models provide the same qualitative results, that is, a quadratic relation in the cohort of subjects with low education and a linear relation in the cohort of subjects with high education. The only exception was the coefficients in mortality from respiratory disease in the cohort of subjects with low education because their significance disappears when moving from independence model to the autoregressive model.

**Fig 2:**
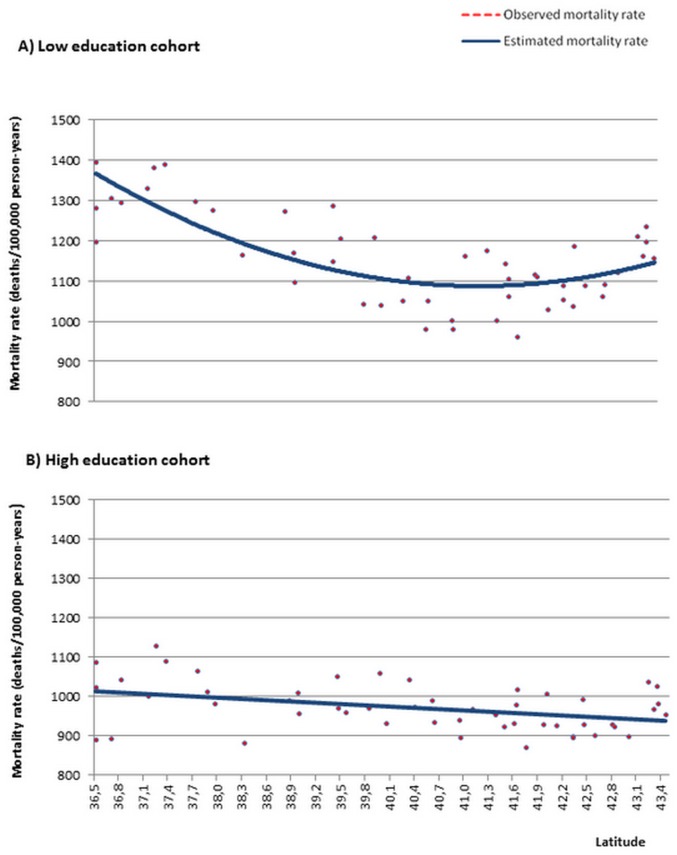
Estimated and observed age- and sex-adjusted mortality rates according to latitude. Spain 2001–2008.

**Fig 3:**
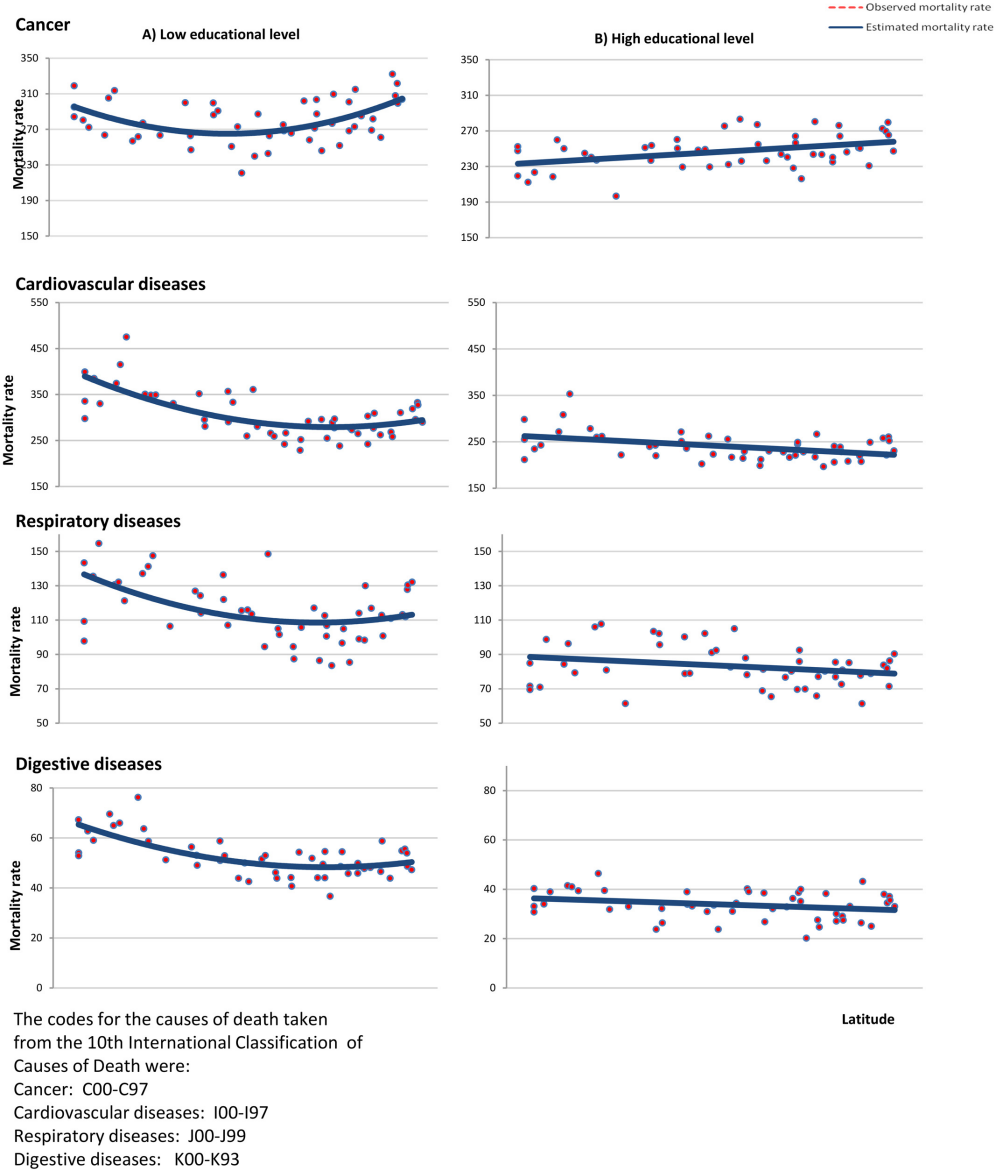
Estimated and observed age- and sex-adjusted mortality rates from several causes of death according to latitude. Spain 2001–2008.

Figs 2 and 3 show the observed and estimated mortality rates as a function of latitude. In the cohort of subjects with low education, the estimated rates were obtained from the quadratic regression models, while in the cohort with high education the estimated rates were obtained from the models using simple linear regression. In the cohort of subjects with low education, the estimated mortality rates have a U shape, such that the lowest rates are seen in the intermediate latitudes. However, while the highest mortality rates from all causes, and from cardiovascular, respiratory and digestive diseases were observed in low latitudes, the highest mortality rates from cancer were seen in higher latitudes. In the cohort of subjects with high education, the estimated mortality rates from all causes, and from cardiovascular, respiratory and digestive diseases decreased from the lowest to the highest latitudes, while the estimated mortality rates for cancer increased.

In the cohort with low education, rurality was associated with reduced mortality from all causes of death analysed, while in the cohort with high education rurality only showed a significant association with mortality from cardiovascular diseases. The findings obtained with and without the variable “rurality” in the regression models are shown in Table 3. In the cohort with low education, the magnitude of the regression coefficients of latitude and of latitude*latitude decreased after including rurality and remained significant only for all-cause mortality, although the coefficients for mortality from cancer and from cardiovascular diseases were marginally significant (p values around 0.07).

**Table 3:** Standardized regression coefficients from regression models predicting provincial sex- and age-adjusted mortality rate in each study cohort for all causes and for leading causes of death. Spain, 2001–2008.

	Low education cohort				High education cohort			
	Model with two independent variables		Model with three independent variables		Model with one independent variable		Model with two independent variables	
	Value	P-value	Value	P-value	Value	P-value	Value	P-value
**All causes**								
Latitude	-19.8	<0.001	-11.3	0.005	-0.39	0.006	-0.32	0.025
Latitude* Latitude	19.2	<0.001	10.8	0.006				
Rurality			-0.4	<0.001			-0.22	0.116
**Cancer**								
Latitude	-20.9	<0.001	-9.2	0.072	0.41	0.003	0.42	0.004
Latitude* Latitude	21.0	<0.001	9.5	0.063				
Rurality			-0.6	<0.001			-0.06	0.684
**Cardiovascular disease**								
Latitude	-15.7	<0.001	-8.9	0.062	-0.43	0.002	-0.35	0.012
Latitude* Latitude	15.1	0.001	8.5	0.077				
Rurality		0.001	-0.3	0.005			-0.28	0.043
**Respiratory disease**								
Latitude	-11.9	0.031	-4.0	0.482	-0.26	0.072	-0.27	0.075
Latitude* Latitude	11.5	0.037	3.7	0.513				
Rurality			-0.4	0.006			0.04	0.779
**Digestive disease**								
Latitude	-14.7	0.002	-8.2	0.096	-0.26	0.070	-0.20	0.182
Latitude* Latitude	14.2	0.003	7.7	0.115				
Rurality			-0.3	0.007			-0.20	0.167

**Fig 4:**
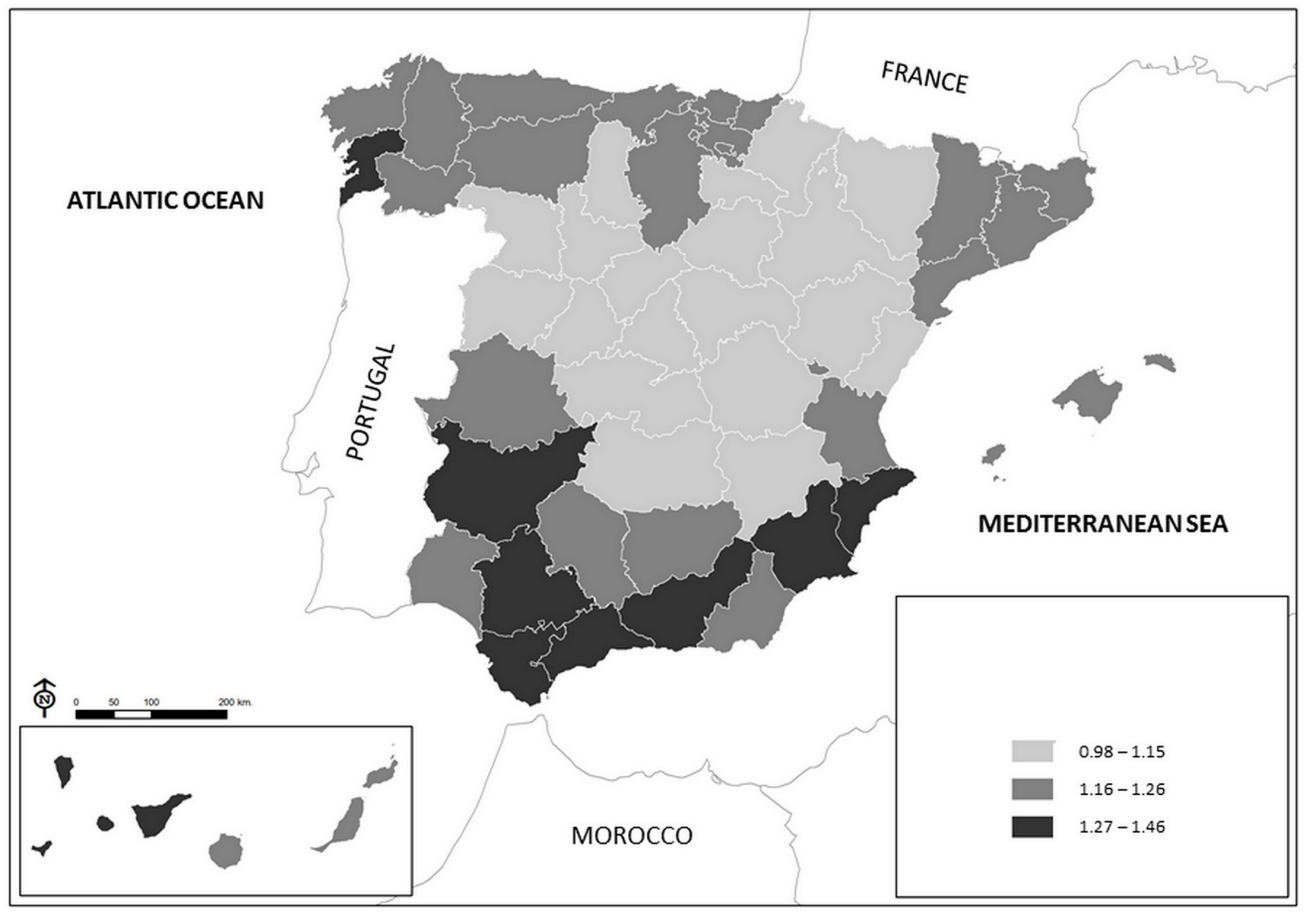
Mortality rate ratio in cohort of subjects with low education versus cohort of subjects with high education, by province. Spain 2001–2008.

The map in Fig 4 shows the mortality rate ratio for all causes of death in the cohort with low education versus the cohort with high education, by province. The highest magnitude is seen in the southern provinces, an intermediate magnitude is observed in the provinces of the north Atlantic coast, the eastern Mediterranean coast and some southern provinces, and the lowest rate ratio is seen in the central provinces and those located in the south of the western Pyrenees.

Table 4 shows the provincial mortality rate observed in cohorts with low and high education and the mortality rate ratio between the two cohorts by latitude intervals. The mortality rate ratio from all causes of death, from the lowest to the highest latitude interval, was 1.27, 1.22, 1.14, 1.16 and 1.20. The pattern of the mortality rate ratio was similar for the leading causes of death analysed, with higher values in the lowest and highest latitudes, and lower values in the intermediate latitudes.

**Table 4:** Sex- and age-adjusted mortality rate per 100,000 person-years in cohort of subjects with low and high education, and mortality rate ratio according to latitude for all causes and for leading causes of death. Spain, 2001–2008.

Cause of death and latitude intervals^1^	Mortality rate^2^		Mortality rate ratio (confidence interval 95%)	
	Low education cohort	High education cohort		
**All causes**				
From 36.53°N to 37.97°N	1299.7	1025.0	1.27	(1.24–1.30)
From 37.98°N to 40.06°N	1187.6	977.4	1.22	(1.18–1.25)
From 40.07°N to 41.60°N	1077.0	942.7	1.14	(1.10–1.19)
From 41.61°N to 42.46°N	1099.4	948.4	1.16	(1.14–1.19)
From 42.47°N to 43.46°N	1155.0	961.9	1.20	(1.18–1.22)
**Cancer**				
From 36.53°N to 37.97°N	285.2	233.3	1.22	(1.18–1.27)
From 37.98°N to 40.06°N	275.1	243.1	1.14	(1.10–1.20)
From 40.07°N to 41.60°N	262.3	252.9	1.04	(0.98–1.11)
From 41.61°N to 42.46°N	285.6	262.9	1.11	(1.08–1.17)
From 42.47°N to 43.46°N	289.7	273.7	1.14	(1.11–1.18)
**Cardiovascular disease**				
From 36.53°N to 37.97°N	370.9	272.5	1.36	(1.30–1.42)
From 37.98°N to 40.06°N	322.8	243.8	1.32	(1.26–1.40)
From 40.07°N to 41.60°N	265.7	219.6	1.21	(1.12–1.32)
From 41.61°N to 42.46°N	283.2	229.4	1.23	(1.16–1.32)
From 42.47°N to 43.46°N	297.3	233.4	1.27	(1.22–1.32)
**Respiratory disease**				
From 36.53°N to 37.97°N	130.3	87.9	1.49	(1.38–1.64)
From 37.98°N to 40.06°N	121.6	89.5	1.35	(1.24–1.50)
From 40.07°N to 41.60°N	111.8	87.1	1.28	(1.13–1.50)
From 41.61°N to 42.46°N	118.5	95.1	1.25	(1.11–1.43)
From 42.47°N to 43.46°N	110.2	73.1	1.50	(1.39–1.64)
**Digestive dsease**				
From 36.53°N to 37.97°N	63.8	38.5	1.65	(1.50–1.86)
From 37.98°N to 40.06°N	52.5	37.8	1.65	(1.45–1.94)
From 40.07°N to 41.60°N	47.3	33.5	1.41	(1.18–1.80)
From 41.61°N to 42.46°N	48.6	31.1	1.56	(1.36–1.88)
From 42.47°N to 43.46°N	50.6	34.4	1.51	(1.38–1.70)

^1^ Latitude intervals reflect the provincial latitude grouped into quintiles.

^2^ The mortality rate reflects the average of the mortality rates in the ten provinces included in each latitude interval.

## Discussion

### Main findings

In the cohort of subjects with low education, the relation of latitude with all-cause mortality and with mortality from the leading causes of death has a U-shaped curve, whereas in the cohort of subjects with high education the relation between latitude and mortality has two different patterns: all-cause mortality and mortality from cardiovascular, respiratory and digestive diseases decreases with increasing latitude, but cancer mortality increases.

At any latitude, the mortality rate for all causes of death analysed is higher in the cohort with low education than in that of high education, although the rate ratio between the two cohorts is higher in the low and high latitudes and lower in the intermediate latitudes. The geographic pattern of the mortality rate ratios is similar to the geographic pattern of the mortality rate in the cohort with low education: the highest magnitude is observed in the southern provinces, an intermediate magnitude in the provinces of the north and the eastern Mediterranean coast, and the lowest magnitude in the central provinces and those in the south of the western Pyrenees.

### Comparison with other studies and possible explanations

Various studies in the Spanish population have observed that residents in areas at lower latitude have the highest rate of all-cause mortality [6,18–19]. The authors of these investigations have suggested that the high mortality in these areas is due to their adverse economic context, since the southern provinces are among those with the lowest per capita income [6,18]. In the present investigation, the two study cohorts also have the highest all-cause mortality in the areas with lowest latitude. However, after adjusting for provincial per capita income in 2001, the magnitude of the relation between latitude and mortality in both cohorts hardly changes (data not shown). This probably occurs because some central provinces have a similar per capita income and show low mortality rates.

The geographic pattern of mortality from all causes in the two cohorts of subjects reflects the geographic pattern observed in mortality from cardiovascular, respiratory and digestive diseases, since these three causes of death are responsible for half of all deaths. A previous study in Spain found a higher prevalence of metabolic syndrome in areas of the south [20]. The authors attributed this finding to obesity and physical inactivity given that the geographic pattern of risk factors is similar to the geographic pattern of metabolic syndrome [21–22]. Since chronic diseases share some risk factors, it is possible that the factors mentioned and other health risk factors are responsible for the geographic pattern observed in mortality from these three causes of death [23].

Both cohorts analysed have the highest cancer mortality in the areas with highest latitude. In Spain higher mortality from various cancer sites has been observed at high latitudes [24]. Similar findings have been seen in studies in France [10] and the United States [25]. There is evidence that ultraviolet radiation and vitamin D reduce the risk of various types of cancer [26]. Since ultraviolet radiation decreases with latitude, some authors have noted that latitude could be an indicator of the dose of ultraviolet radiation and, consequently, of vitamin D production [27].

The findings of this study also show that the factors responsible for increased mortality in the cohort of subjects with low educational level, with respect to the cohort of subjects with high educational level, are in any latitude in Spain, although the magnitude of the difference in mortality between the two cohorts shows a geographic pattern. Two previous investigations in Italy and the United States have studied the geographic pattern of inequalities in mortality [12–13]. In Italy, the mortality rate ratio by education is higher in regions of the south [12], whereas in the United States the mortality rate ratio does not vary from one region to another [13]. This could be attributed to the fact that in Italy the geographic pattern of mortality varies by education, whereas in the United States it does not. In Spain, the relation between latitude and mortality is linear in the cohort with high education, whereas in the cohort with low education the lowest mortality is observed at intermediate latitudes. As a result, the mortality rate ratio by education is higher in the south and north, and lower in the central region.

Why is the relation between latitude and mortality U-shaped in the cohort with low education? That is, why does the cohort with low education in the provinces in the large territory making up central Spain–which is economically heterogeneous–have low mortality? The explanation may be the high percentage of the population residing in rural areas in these provinces, since this characteristic is associated with reduced mortality from all the causes of death studied. However, for mortality from cancer and cardiovascular diseases there must be an additional explanation. After including rurality, the regression coefficients of latitude and latitude*latitude were on the margins of statistical significance. In fact, the regression coefficients for all-cause mortality remained significant after including rurality, given that deaths from these two causes represent 50% of all deaths. In any case, the provinces included within each latitude quintile do not exhibit the heterogeneity in rurality observed in the analysis carried out with all provinces and, therefore, the magnitude of the mortality rate ratio in each quintile was not changed when the rurality was included as adjustment variable.

Altitude may be the additional explanation for this lower mortality from cancer and cardiovascular diseases in the central provinces. These provinces are located in a large central massif, known as the “Meseta Central”, which occupies almost half of the country’s surface area. The main characteristic of the Meseta is its mean altitude (between 600 and 700 m), with the highest altitude in the northern sub-plateau [28]. Studies in a number of countries have found that high areas have lower mortality from different cancer sites, ischaemic heart disease and cerebrovascular disease [29–34]. Likewise, it may be argued that moderate altitudes are more protective than high or even very high altitudes [35]. The immediate question is why residence in high areas could show a protective effect on health in a cohort of subjects with low education, but not in those with high education. The answer must be sought in the low mortality rates in the cohort of subjects with high education throughout the entire country. Given a characteristic of the physical environment, in this case, altitude, which probably reduces the mortality risk with respect to other areas, the margin for reduction is smaller in persons with high education, who have the lowest mortality rates.

Finally, since the level of education is a proxy for socioeconomic status, it can not be ruled out that among the subjects of the cohort with low education, those who live in areas of high altitude present a higher frequency of other socioeconomic characteristics associated with health than those living in other parts of Spain. For example, if areas in high altitude offer healthier occupations or occupations that generate higher incomes this circumstance may help explain the protective effect of altitude on those individuals.

### Strengths and limitations

This study has no problems of representativeness since it included all adults age 25 and over who resided in the Spanish provinces at the time of the 2001 population census. Furthermore, as compared to previous investigations of the geographic pattern of mortality in only three areas–northern, mid and southern Italy, [12]–or in four areas–the Northeast, Midwest, South and West of the United States [13]–the present study has analysed 50 geographic areas. The two aforementioned studies showed a geographic pattern of inequalities only in all-cause mortality, however, the present investigation also estimated the geographic pattern of inequalities in mortality from the leading causes of death.

Subjects in the two study cohorts who resided in the two provinces of the Canary Islands were included in the analysis, although they were assigned the latitude of the southernmost province of the Iberian peninsula. In any case, excluding residents of these two provinces from the analysis did not change the results. Moreover, to simplify the presentation of results, the mortality rates were adjusted for age and sex. We previously ruled out the existence of interaction by sex in both study cohorts in the relation between latitude and the age-adjusted mortality rates.

Education was used as the indicator of socioeconomic position. Therefore, it is not possible to know what the geographic pattern would be using other socioeconomic indicators like income or social class based on occupation, because income was not collected in the census and occupation was registered only in subjects who were employed in the week before the census. Nonetheless, education has the advantage of allowing classification of all subjects. Furthermore, use of this indicator allowed comparison with the only two studies that have analysed geographic patterns in mortality inequalities.

In conclusion, inequalities in mortality by education in Spain are highest in the north and south of the country, and lowest in the large central region of Spain that makes up the Meseta. This geographic pattern reflects the mortality pattern observed in the cohort of subjects with low education. Rurality and the greater frequency of socioeconomic characteristics that protect health may explain the low mortality in subjects with low education who live in that area.

## Supporting Information

S1 Table Characteristics distribution of study subjects by province. Spain 2001–2008.
(DOCX)

S2 Table Linear regression models fitted by including latitude or longitude. Parameter estimates and p-values from models predicting provincial sex- and age-adjusted mortality rate in each study cohort for all causes and for leading causes of death. Spain, 2001–2008.
(DOCX)

S3 Table Quadratic regression models fitted by including latitude and latitude*latitude. Parameter estimates and p-values from models predicting provincial sex- and age-adjusted mortality rate in each study cohort for all causes and for leading causes of death. Spain, 2001–2008.
(DOCX)

S4 Table Comparison of a standard regression [independence model] and an autoregressive model [simultaneous autoregressive model]. (DOCX)
