# Supplementary material for: The Association of Geographic Coordinates with Mortality in People with Lower and Higher Education and with Mortality Inequalities in Spain
Source: PLoS One. 2015 Jul 24;10(7):e0133765. doi: 10.1371/journal.pone.0133765 (PMC4514891; doi:10.1371/journal.pone.0133765)
Supplement: S1 Table — Spain 2001–2008. (DOCX) [file pone.0133765.s001.docx]

| **Table S1. Characteristics distribution of study subjects by province. Spain 2001-2008.** | | | | | | | |
| --- | --- | --- | --- | --- | --- | --- | --- |
| **Province** | **Persons-year at risk** | **Age (mean)** | **Gender (%women)** | **Rurality (1)** | **Educational level (2)** | **Unadjusted overall mortality rate (3)** | **Adjusted overall mortality rate (4)** |
|  |  |  |  |  |  |  |  |
| Alava | 1,456,449 | 49 | 51.0 | 14.5 | 40.7 | 1031 | 1050 |
| Albacete | 1,704,388 | 51 | 51.1 | 29.8 | 23.1 | 1246 | 1080 |
| Alicante | 7,005,667 | 50 | 51.4 | 6.4 | 26.9 | 1134 | 1130 |
| Almería | 2,371,669 | 48 | 50.6 | 18.8 | 25.2 | 1142 | 1260 |
| Avila | 827,771 | 54 | 50.1 | 63.0 | 22.5 | 1558 | 1030 |
| Badajoz | 3,011,341 | 51 | 51.3 | 33.5 | 22.0 | 1391 | 1250 |
| Baleares | 4,033,504 | 49 | 51.0 | 9.7 | 31.6 | 1138 | 1160 |
| Barcelona | 23,326,928 | 50 | 52.4 | 5.2 | 36.2 | 1188 | 1130 |
| Burgos | 1,748,999 | 52 | 50.3 | 33.0 | 33.7 | 1291 | 1010 |
| Cáceres | 1,777,000 | 52 | 51.0 | 49.9 | 21.5 | 1574 | 1130 |
| Cádiz | 5,012,787 | 48 | 51.4 | 2.3 | 27.3 | 1097 | 1350 |
| Castellón | 2,387,312 | 50 | 50.9 | 22.7 | 25.4 | 1314 | 1190 |
| Ciudad Real | 2,238,605 | 51 | 51.9 | 22.1 | 20.5 | 1405 | 1150 |
| Córdoba | 3,496,498 | 50 | 52.2 | 15.1 | 23.5 | 1297 | 1200 |
| Coruña (La) | 5,522,447 | 51 | 53.2 | 10.5 | 29.5 | 1355 | 1140 |
| Cuenca | 976,257 | 54 | 50.6 | 57.4 | 18.4 | 1556 | 1040 |
| Girona | 2,870,784 | 50 | 50.8 | 33.7 | 30.0 | 1214 | 1090 |
| Granada | 3,715,868 | 50 | 52.0 | 26.9 | 29.0 | 1270 | 1250 |
| Guadalajara | 946,441 | 50 | 49.3 | 50.2 | 33.1 | 1158 | 970 |
| Guipúzcoa | 3,395,759 | 50 | 51.8 | 10.7 | 40.1 | 1163 | 1110 |
| Huelva | 2,105,183 | 49 | 51.3 | 21.9 | 23.7 | 1310 | 1330 |
| Huesca | 1,041,102 | 53 | 49.9 | 44.9 | 29.7 | 1488 | 1030 |
| Jaén | 2,891,126 | 51 | 51.6 | 22.9 | 21.2 | 1363 | 1240 |
| León | 2,460,178 | 54 | 52.0 | 44.0 | 28.4 | 1484 | 1050 |
| Lleida | 1,807,893 | 52 | 50.6 | 48.2 | 30.8 | 1455 | 1110 |
| Rioja (La) | 1,375,376 | 51 | 50.7 | 29.1 | 33.0 | 1223 | 1030 |
| Lugo | 1,846,940 | 55 | 52.1 | 39.6 | 22.0 | 1738 | 1090 |
| Madrid | 26,077,359 | 49 | 53.1 | 3.2 | 44.4 | 972 | 1040 |
| Málaga | 5,976,355 | 48 | 51.9 | 11.3 | 29.9 | 1127 | 1240 |
| Murcia | 5,430,801 | 48 | 51.2 | 1.4 | 28.0 | 1144 | 1220 |
| Navarra | 2,742,132 | 50 | 50.9 | 37.6 | 37.0 | 1167 | 1040 |
| Orense | 1,727,611 | 55 | 53.0 | 46.0 | 21.3 | 1712 | 1060 |
| Asturias | 5,457,974 | 52 | 53.0 | 8.6 | 32.4 | 1476 | 1190 |
| Palencia | 851,692 | 53 | 51.2 | 42.9 | 28.8 | 1418 | 1080 |
| Palmas (Las) | 4,076,775 | 46 | 50.3 | 2.0 | 31.5 | 918 | 1230 |
| Pontevedra | 4,359,078 | 50 | 53.2 | 8.8 | 26.5 | 1240 | 1140 |
| Salamanca | 1,710,958 | 53 | 52.3 | 40.3 | 29.7 | 1376 | 960 |
| S. C. de Tenerife | 3,779,713 | 48 | 51.2 | 8.0 | 30.7 | 1000 | 1150 |
| Cantabria | 2,709,368 | 51 | 52.1 | 23.9 | 34.3 | 1265 | 1110 |
| Segovia | 728,403 | 53 | 50.4 | 50.0 | 30.0 | 1402 | 990 |
| Sevilla | 7,916,418 | 48 | 52.1 | 7.1 | 29.4 | 1185 | 1340 |
| Soria | 455,619 | 55 | 50.0 | 56.8 | 28.1 | 1516 | 940 |
| Tarragona | 3,136,392 | 50 | 50.7 | 27.1 | 30.1 | 1246 | 1130 |
| Teruel | 677,528 | 55 | 49.6 | 63.4 | 22.2 | 1623 | 1050 |
| Toledo | 2,712,117 | 50 | 50.5 | 48.7 | 21.7 | 1239 | 1060 |
| Valencia | 10,745,306 | 50 | 52.0 | 12.2 | 31.6 | 1253 | 1250 |
| Valladolid | 2,293,477 | 50 | 51.7 | 23.4 | 35.9 | 1141 | 1050 |
| Vizcaya | 5,680,345 | 51 | 52.4 | 10.1 | 40.1 | 1220 | 1160 |
| Zamora | 1,011,098 | 55 | 51.2 | 56.2 | 20.9 | 1635 | 980 |
| Zaragoza | 4,305,778 | 51 | 51.8 | 18.8 | 34.5 | 1319 | 1110 |
|  |  |  |  |  |  |  |  |
| 1) Percentage of people living in municipalities with 5,000 or less inhabitants. | | | | | | | |
| 2) Percentage of people with upper secondary studies or higher. | | | | | | | |
| 3) Mortality rate per 100,000 person-years at risk. | | | | | | | |
| 4) Sex- and age-adjusted mortality rate per 100,000 person-years at risk. | | | | | | | |
