# Supplementary material for: The Association of Geographic Coordinates with Mortality in People with Lower and Higher Education and with Mortality Inequalities in Spain
Source: PLoS One. 2015 Jul 24;10(7):e0133765. doi: 10.1371/journal.pone.0133765 (PMC4514891; doi:10.1371/journal.pone.0133765)
Supplement: S2 Table — Parameter estimates and p-values from models predicting provincial sex- and age-adjusted mortality rate in each study cohort for all causes and for leading causes of death. Spain, 2001–2008. (DOCX) [file pone.0133765.s002.docx]

| Table S2. Linear regression models fitted by including latitude or longitude. Parameter estimates and p-values from models predicting provincial sex- and age-adjusted mortality rate in each study cohort for all causes and for leading causes of death. Spain, 2001-2008. | | | | | | | | |
| --- | --- | --- | --- | --- | --- | --- | --- | --- |
|  |  |  |  |  |  |  |  |  |
|  |  |  |  |  |  |  |  |  |
|  |  |  |  |  |  |  |  |  |
|  |  | Low education cohort | | |  | High education cohort | | |
|  |  |  |  |  |  |  |  |  |
|  |  | Coefficient |  | P-value |  | Coefficient |  | P-value |
| **All causes** |  |  |  |  |  |  |  |  |
| Intercept |  | 2444.4 |  | <0.001 |  | 1408.4 |  | <0.001 |
| Latitude |  | -31.9 |  | <0.001 |  | -10.8 |  | 0.006 |
|  |  |  |  |  |  |  |  |  |
| **Cancer** |  |  |  |  |  |  |  |  |
| Intercept |  | 226.9 |  | <0.001 |  | 103.6 |  | 0.031 |
| Latitude |  | 1.3 |  | 0.410 |  | 3.5 |  | 0.003 |
|  |  |  |  |  |  |  |  |  |
| **Cardiovascular disease** | |  |  |  |  |  |  |  |
| Intercept |  | 859.6 |  | <0.001 |  | 475.4 |  | <0.001 |
| Latitude |  | -13.7 |  | <0.001 |  | -5.8 |  | 0.002 |
|  |  |  |  |  |  |  |  |  |
| **Respiratory disease** | |  |  |  |  |  |  |  |
| Intercept |  | 252.1 |  | <0.001 |  | 140.0 |  | <0.001 |
| Latitude |  | -3.4 |  | 0.002 |  | -1.4 |  | 0.072 |
|  |  |  |  |  |  |  |  |  |
| **Digestive disease** | |  |  |  |  |  |  |  |
| Intercept |  | 139.9 |  | <0.001 |  | 31.3 |  | <0.001 |
| Latitude |  | -2.2 |  | <0.001 |  | -0.7 |  | 0.070 |
|  |  |  |  |  |  |  |  |  |
|  |  |  |  |  |  |  |  |  |
| **All causes** |  |  |  |  |  |  |  |  |
| Intercept |  | 1132.2 |  | <0.001 |  | 969.6 |  | <0.001 |
| Longitude |  | -6.3 |  | 0.245 |  | -0.7 |  | 0.821 |
|  |  |  |  |  |  |  |  |  |
| **Cancer** |  |  |  |  |  |  |  |  |
| Intercept |  | 272.5 |  | <0.001 |  | 243.7 |  | <0.001 |
| Longitude |  | -2.1 |  | 0.072 |  | -0.9 |  | 0.323 |
|  |  |  |  |  |  |  |  |  |
| **Cardiovascular disease** | |  |  |  |  |  |  |  |
| Intercept |  | 294.7 |  | <0.001 |  | 231.7 |  | <0.001 |
| Longitude |  | -3.0 |  | 0.217 |  | -2.2 |  | 0.116 |
|  |  |  |  |  |  |  |  |  |
| **Respiratory disease** | |  |  |  |  |  |  |  |
| Intercept |  | 111.6 |  | <0.001 |  | 81.0 |  | <0.001 |
| Longitude |  | -1.0 |  | 0.228 |  | -0.6 |  | 0.313 |
|  |  |  |  |  |  |  |  |  |
| **Digestive disease** | |  |  |  |  |  |  |  |
| Intercept |  | 52.0 |  | <0.001 |  | 33.8 |  | <0.001 |
| Longitude |  | -0.1 |  | 0.795 |  | 0.1 |  | 0.858 |
