# Supplementary material for: The Association of Geographic Coordinates with Mortality in People with Lower and Higher Education and with Mortality Inequalities in Spain
Source: PLoS One. 2015 Jul 24;10(7):e0133765. doi: 10.1371/journal.pone.0133765 (PMC4514891; doi:10.1371/journal.pone.0133765)
Supplement: S3 Table — Parameter estimates and p-values from models predicting provincial sex- and age-adjusted mortality rate in each study cohort for all causes and for leading causes of death. Spain, 2001–2008. (DOCX) [file pone.0133765.s003.docx]

| Table S3. Quadratic regression models fitted by including latitude and latitude*latitude. Parameter estimates and p-values from models predicting provincial sex- and age-adjusted mortality rate in each study cohort for all causes and for leading causes of death. Spain, 2001-2008. | | | | | | | | |
| --- | --- | --- | --- | --- | --- | --- | --- | --- |
|  |  |  |  |  |  |  |  |  |
|  |  |  |  |  |  |  |  |  |
|  |  |  |  |  |  |  |  |  |
|  |  | Low education cohort | | |  | High education cohort | | |
|  |  |  |  |  |  |  |  |  |
|  |  | Coefficient |  | P-value |  | Coefficient |  | P-value |
| **All causes** |  |  |  |  |  |  |  |  |
| Intercept |  | 22179.7 |  | <0.001 |  | 338.9 |  | 0.291 |
| Latitude |  | -1021.8 |  | <0.001 |  | -109.7 |  | 0.493 |
| Latitude* Latitude | | 12.4 |  | <0.001 |  | 1.2 |  | 0.536 |
|  |  |  |  |  |  |  |  |  |
| **Cancer** |  |  |  |  |  |  |  |  |
| Intercept |  | 4847.2 |  | <0.001 |  | 167.8 |  | 0.865 |
| Latitude |  | -230.4 |  | <0.001 |  | 0.3 |  | 0.994 |
| Latitude* Latitude | | 2.9 |  | <0.001 |  | 0.1 |  | 0.948 |
|  |  |  |  |  |  |  |  |  |
| **Cardiovascular disease** | |  |  |  |  |  |  |  |
| Intercept |  | 7757.8 |  | <0.001 |  | 3215.3 |  | 0.032 |
| Latitude |  | -359.7 |  | <0.001 |  | -143.3 |  | 0.056 |
| Latitude* Latitude | | 4.3 |  | 0.001 |  | 1.7 |  | 0.066 |
|  |  |  |  |  |  |  |  |  |
| **Respiratory disease** | |  |  |  |  |  |  |  |
| Intercept |  | 2088.9 |  | 0.018 |  | -861.1 |  | 0.184 |
| Latitude |  | -95.5 |  | 0.031 |  | 48.8 |  | 0.135 |
| Latitude* Latitude | | 1.2 |  | 0.037 |  | -0.6 |  | 0.124 |
|  |  |  |  |  |  |  |  |  |
| **Digestive disease** | |  |  |  |  |  |  |  |
| Intercept |  | 1164.4 |  | <0.001 |  | 442.9 |  | 0.160 |
| Latitude |  | -53.6 |  | 0.002 |  | -19.8 |  | 0.208 |
| Latitude* Latitude | | 0.6 |  | 0.003 |  | 0.2 |  | 0.224 |
|  |  |  |  |  |  |  |  |  |
