# Supplementary material for: The Association of Geographic Coordinates with Mortality in People with Lower and Higher Education and with Mortality Inequalities in Spain
Source: PLoS One. 2015 Jul 24;10(7):e0133765. doi: 10.1371/journal.pone.0133765 (PMC4514891; doi:10.1371/journal.pone.0133765)
Supplement: S4 Table — (DOCX) [file pone.0133765.s004.docx]

| Table S4. Comparison of a standard regression (independence model) and an autoregressive model (simultaneous autoregressive model) | | | | | | | | | | | | | | | | | | | | |
| --- | --- | --- | --- | --- | --- | --- | --- | --- | --- | --- | --- | --- | --- | --- | --- | --- | --- | --- | --- | --- |
|  |  | **Low education cohort** | | | | | | | | |  | **High education cohort** | | | | | | | | |
|  |  | -2 Res Log Likelihood |  | Latitude | | |  | Latitude*Latitude | | |  | -2 Res Log Likelihood |  | Latitude | | |  | Latitude*Latitude | | |
|  |  |  |  |  |  |  |  |  |  |  |  |  |  |  |  |  |  |  |  |  |
|  |  |  |  | Coefficient |  | p-value |  | Coefficient |  | p-value |  |  |  | Coefficient |  | p-value |  | Coefficient |  | p-value |
|  |  |  |  |  |  |  |  |  |  |  |  |  |  |  |  |  |  |  |  |  |
| **All causes** |  |  |  |  |  |  |  |  |  |  |  |  |  |  |  |  |  |  |  |  |
| Independence model | | 554.0 |  | -1021.8 |  | <0.001 |  | 12.4 |  | <0.001 |  | 529.9 |  | -109.7 |  | 0.493 |  | 1.2 |  | 0.536 |
| Simultaneous autoregressive model | | 557.7 |  | -1027.2 |  | <0.001 |  | 12.4 |  | <0.001 |  | 533.7 |  | -103.6 |  | 0.526 |  | 1.2 |  | 0.569 |
|  |  |  |  |  |  |  |  |  |  |  |  |  |  |  |  |  |  |  |  |  |
| **Cancer** |  |  |  |  |  |  |  |  |  |  |  |  |  |  |  |  |  |  |  |  |
| Independence model | | 436.4 |  | -230.4 |  | <0.001 |  | 2.9 |  | <0.001 |  | 420.0 |  | 0.3 |  | 0.994 |  | 0.1 |  | 0.948 |
| Simultaneous autoregressive model | | 439.1 |  | -249.3 |  | <0.001 |  | 3.1 |  | <0.001 |  | 423.3 |  | 2.5 |  | 0.960 |  | 0.0 |  | 0.985 |
|  |  |  |  |  |  |  |  |  |  |  |  |  |  |  |  |  |  |  |  |  |
| **Cardiovascular disease** | |  |  |  |  |  |  |  |  |  |  |  |  |  |  |  |  |  |  |  |
| Independence model | | 488.1 |  | -359.7 |  | <0.001 |  | 4.3 |  | 0.001 |  | 456.9 |  | -143.3 |  | 0.056 |  | 1.7 |  | 0.066 |
| Simultaneous autoregressive model | | 489.1 |  | -298.3 |  | 0.010 |  | 3.6 |  | 0.010 |  | 459.3 |  | -130.8 |  | 0.096 |  | 1.6 |  | 0.110 |
|  |  |  |  |  |  |  |  |  |  |  |  |  |  |  |  |  |  |  |  |  |
| **Respiratory disease** | |  |  |  |  |  |  |  |  |  |  |  |  |  |  |  |  |  |  |  |
| Independence model | | 406.7 |  | -95.5 |  | 0.031 |  | 1.2 |  | 0.037 |  | 379.5 |  | 48.8 |  | 0.135 |  | -0.6 |  | 0.124 |
| Simultaneous autoregressive model | | 405.7 |  | -74.6 |  | 0.091 |  | 0.9 |  | 0.103 |  | 378.9 |  | 45.0 |  | 0.160 |  | -0.6 |  | 0.148 |
|  |  |  |  |  |  |  |  |  |  |  |  |  |  |  |  |  |  |  |  |  |
| **Digestive disease** | |  |  |  |  |  |  |  |  |  |  |  |  |  |  |  |  |  |  |  |
| Independence model | | 316.7 |  | -53.6 |  | 0.002 |  | 0.6 |  | 0.003 |  | 311.4 |  | -19.8 |  | 0.208 |  | 0.2 |  | 0.224 |
| Simultaneous autoregressive model | | 318.2 |  | -46.3 |  | 0.012 |  | 0.6 |  | 0.012 |  | 313.0 |  | -21.2 |  | 0.186 |  | 0.3 |  | 0.203 |
|  |  |  |  |  |  |  |  |  |  |  |  |  |  |  |  |  |  |  |  |  |
|  |  |  |  |  |  |  |  |  |  |  |  |  |  |  |  |  |  |  |  |  |
